# Analysis of Whole-Genome as a Novel Strategy for Animal Species Identification

**DOI:** 10.3390/ijms25052955

**Published:** 2024-03-03

**Authors:** Yutong Gan, Guihong Qi, Lijun Hao, Tianyi Xin, Qian Lou, Wenjie Xu, Jingyuan Song

**Affiliations:** 1 Key Lab of Chinese Medicine Resources Conservation, State Administration of Traditional Chinese Medicine of the People’s Republic of China, Institute of Medicinal Plant Development, Chinese Academy of Medical Sciences & Peking Union Medical College, Beijing 100193, China; keira1106@163.com (Y.G.); qiguihong12@163.com (G.Q.); haolj_cpu@foxmail.com (L.H.); tyxin@implad.ac.cn (T.X.); louq97@126.com (Q.L.); 2 Engineering Research Center of Chinese Medicine Resource, Ministry of Education, Beijing 100193, China

**Keywords:** Analysis of whole-GEnome, bioinformatics analysis, animal species identification, sanger sequencing, CRISPR-Cas12a

## Abstract

Survival crises stalk many animals, especially endangered and rare animals. Accurate species identification plays a pivotal role in animal resource conservation. In this study, we developed an animal species identification method called Analysis of whole-GEnome (AGE), which identifies species by finding species-specific sequences through bioinformatics analysis of the whole genome and subsequently recognizing these sequences using experimental technologies. To clearly demonstrate the AGE method, *Cervus nippon*, a well-known endangered species, and a closely related species, *Cervus elaphus*, were set as model species, without and with published genomes, respectively. By analyzing the whole genomes of *C. nippon* and *C. elaphus*, which were obtained through next-generation sequencing and online databases, we built specific sequence databases containing 7,670,140 and 570,981 sequences, respectively. Then, the species specificities of the sequences were confirmed experimentally using Sanger sequencing and the CRISPR-Cas12a system. Moreover, for 11 fresh animal samples and 35 commercially available products, our results were in complete agreement with those of other authoritative identification methods, demonstrating AGE’s precision and potential application. Notably, AGE found a mixture in the 35 commercially available products and successfully identified it. This study broadens the horizons of species identification using the whole genome and sheds light on the potential of AGE for conserving animal resources.

## 1. Introduction

Biodiversity is one of the most precious treasures of life on Earth [1] and also a tremendous challenge. Each species plays a unique role and interacts with other species, forming intricate ecological connections. The accurate identification of species is crucial for understanding, conserving, and managing ecosystems on Earth. To distinguish various species, multiple identification methods have been developed. Currently, morphological identification remains the most fundamental and widely recognized method. This approach involves experts with extensive specialized knowledge identifying species’ traits, which are susceptible to environmental factors and intraspecific variation. Physical and chemical identification extends our understanding of a species beyond its morphological characteristics to encompass its distinct material composition. This form of species identification typically necessitates standard controls. In addition to morphological identification and physical and chemical identification, nucleic acid-based identification methods have been carried out with the progress of molecular technologies [2,3,4,5]. DNA barcoding is currently the most widely used molecular identification method [6,7,8,9]. The successive unraveling of genomes and increasing developments in bioinformatics have enabled us to propose a novel method of species identification in this paper: Analysis of whole-GEnome (AGE) [10,11,12]. AGE is a molecular diagnostic method that searches for species-specific sequences from the whole genome of the target species, accurately identifies species-specific sequences, and achieves accurate species identification.

Since its launch, sequencing technology has completed two upgrades from first-generation sequencing technology, Sanger sequencing [13], to next-generation sequencing (NGS) technology, and then to third-generation sequencing (TGS) technology [14]. The iteration of sequencing technologies has made sequencing species genomes affordable for most researchers, providing a strong impetus for the rapid development of genomics. The Earth Biogenome Project is dedicated to deciphering the genomes of the nearly 1.5 million eukaryotic species known on Earth [15]. The rapid development of sequencing technology and the increasing focus on genomes have contributed to the explosive increase in the number of species whose genomes are publicly available. According to the National Center for Biotechnology Information (NCBI), there were 30,398 eukaryotic genomes publicly available as of June 2023. The rapid development of bioinformatics has become an important driver of modern life science research. With the rendezvous of multidisciplinary knowledge from computer science, statistics, and biology, bioinformatics provides us with powerful tools for processing and analyzing large-scale biological data. With the continuous advancement of computer technology and optimization of algorithms, bioinformatics has become more and more efficient, accurate, and reliable. The boom in the development of technologies for acquiring and analyzing genomes supports AGE in achieving species identification.

8.74 million eukaryote species live on Earth, and more than three-quarters of these belong to the animal kingdom [16]. Abundant animal resources, whether for food [17], medicine [18], or industry [19], are critical to human survival. Nevertheless, biodiversity loss in the animal kingdom was severe during the “sixth extinction wave” [1]. According to the Worldwide Fund For Nature, many animals, including Javan rhinos, Amur leopards, and Yangtze finless porpoises, are facing serious threats to their survival due to habitat destruction and over-hunting. Meanwhile, the illegal trade of endangered and rare animals is quite a serious problem. Accurate animal species identification underpins animal resource conservation and animal biodiversity. Implementing animal species identification through AGE not only solves the problem of animal species identification but also helps maintain the stability of ecosystems and protects species and genetic diversity.

In this paper, to establish an AGE identification system in the animal field, *Cervus nippon* and *Cervus elaphus* were selected as model species. *C. nippon* belongs to the *Cervidae* family and is a rare species in China. It is listed as a Class I protected species in China and was included in the International Union for Conservation of Nature (IUCN) 2015 Red List of Endangered Species. *C. elaphus* is a closely related species to *C. nippon* and is often encountered as a common substitute for, or misidentified as, *C. nippon*. We used *C. elaphus* and *C. nippon* as model species with and without publicly available genomes, respectively, to illustrate the process of analyzing the whole genome of a species via bioinformatics. There were various ways for AGE to precisely identify species-specific target sequences, and the Sanger sequencing and CRISPR-Cas12a system were chosen as experimental technologies in this paper. The fluorescence presentation of the CRISPR-Cas12a system consists of three forms: fluorescence values, visual fluorescence, and lateral flow tests. AGE successfully achieved the identification of animal samples from *C. nippon*, *C. elaphus*, and *Rangifer tarandus*, as well as the identification of animal species components in commercially available products and exhibited good performance. In this study, an accurate and effective method of identification has been created in the animal kingdom, called AGE. It effectively identified closely related species, providing an example for the identification of other closely related species, and offers a new approach to the regulation of commercial products and the protection of endangered species.

## 2. Results

### 2.1. Screening for Species-Specific Sequences through Bioinformatics Analysis of the Whole Genome

The initial stage of species identification using AGE involves utilizing bioinformatics to discover species-specific sequences from its genome. According to whether the target species genome was publicly available or not, we elaborated on the detailed steps for undertaking bioinformatics analysis to identify species-specific sequences in these two different cases.

Since the genome of *C. elaphus* has been published, we developed the AGE system and employed *C. elaphus* as a model species. We initially extracted random fragments from the genome of *C. elaphus* to form a fragment sequence database. Subsequently, we selected sequences from the fragment sequence database that contained the protospacer adjacent motif (PAM) to construct a candidate fragment sequence database. The analysis revealed that the fragment sequence database contained nearly 2.1 billion sequences, while the number of candidate fragment sequences was less than 5% of the fragment sequences (Table S1). Finally, we aligned the candidate target sequences of *C. elaphus* with the fragment sequence databases of *C. nippon* and *R. tarandus*. We selected sequences from the sequence alignment results that showed three or more mismatches and all indels within 21 bp, excluding the PAM sequence. Nearly 570,000 sequences were used to construct the specific target sequence database for *C. elaphus*. We randomly selected two species-specific target sequences located in the nuclear genome and another located in the mitochondrial genome, named Ce_target1, Ce_target2, and Ce_target3, respectively (Table 1). The species-specificity of specific sequences can be further validated extensively across a wide range of species by blasting them with all the genomes in the NCBI database. This enables their application across a broad spectrum of species.

Based on the aforementioned three target sequences, we designed crRNAs (CRISPR RNAs) for the CRISPR-Cas system. Additionally, we designed universal primers based on the upstream and downstream sequences of the target sequences. These universal primers serve the purpose of sequencing and amplification in further experiments. The specific sequences of crRNAs and universal primers, corresponding to each target sequence, are summarized in Table 1 and Table S2.

During the construction of the AGE system, as the genome of *C. nippon* species was not publicly available, we employed it as a model species to demonstrate how the AGE system could be established in the absence of access to the specific genome of the target species. The primary goal of AGE was to obtain the genomes of target species and their related species. We applied shallow sequencing technology to obtain the genome of *C. nippon*. First, we collected a fresh sample of *C. nippon* meat (Table S3). Subsequently, we performed next-generation sequencing (NGS) on this sample, generating 199.7 Gb of shotgun whole genome sequencing (sWGS) data. 

The bioinformatic analysis of the sWGS data of *C. nippon* was performed in the same way as that of the genome of *C. elaphus*. Bioinformatics analysis of the genome of *C. nippon* revealed that the fragment sequence database contained more than 3 billion sequences, the candidate fragment sequence database included more than 158 million sequences, and the specific target sequence database consisted of nearly 7.7 million sequences (Table S1). We also randomly selected one species-specific target sequence located in the mitochondrial genome and two in the nuclear genome, named Cn_target1, Cn_target2, and Cn_target3, respectively. 

In 2022, high-quality, chromosome-level genomes of *C. nippon* were published [20,21]. To investigate the effect of genome quality on the results of bioinformatic analysis, the sWGS data of *C. nippon* was compared with a published *C. nippon* genome. The published genome was assembled from approximately 269 Gb of data, while we obtained a total of 199.7 Gb of clean data. To examine the generality and specificity of the specific target sequences found from the sWGS data of *C. nippon*, we analyzed the predicted off-targets of Cn_target1 and Cn_target2 by mapping them to the published genomes of *C. nippon*, *C. elaphus*, and *R. tarandus*. The prediction showed that there was no sequence within the three base mismatches compared to Cn_target1 and Cn_target2 in the genome of *C. elaphus* and *R. tarandus*, and one interesting finding was that no exact match to Cn_target1 could be found in the published genome, but there was one sequence with one base mismatch (Tables S4 and S5). More interestingly, all three of the fresh *C. nippon* animal samples contained Cn_target1, according to the results of the latter experiment. We reason that this is most likely due to intraspecific variation in the species. Moreover, the intraspecific variation that differs from the species-specific sequence by one base does not affect the identification results of AGE. The results demonstrated that the sWGS data was sufficient for the bioinformatics analysis of AGE.

### 2.2. Species Identification by Discerning Species-Specific Sequences Using Different Experimental Technologies

**Figure 1:**
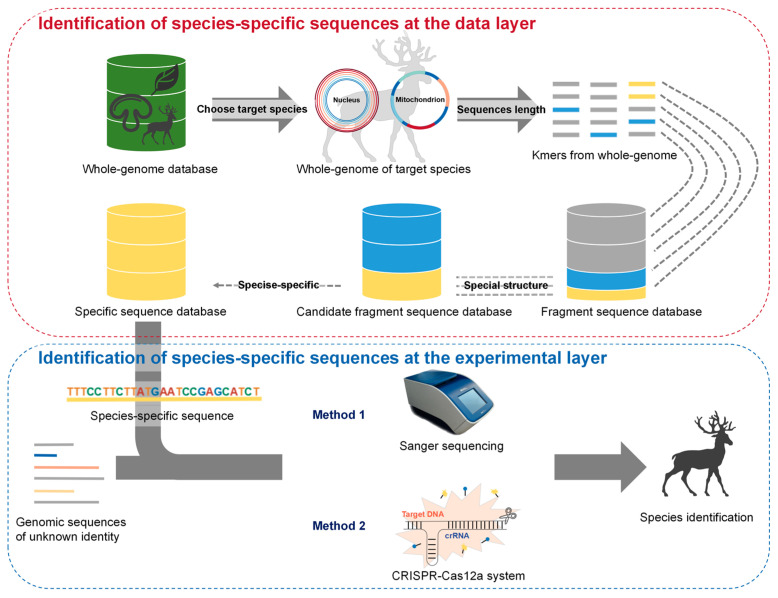
Proposed workflow to implement Analysis of whole-GEnome (AGE) for species identification. AGE contains two key steps: bioinformatics analysis and experimental operations. Bioinformatics analysis is applied to identify the species-specific sequences of each species. The whole genome database contains the whole genome sequences of all eukaryotic species. Bioinformatics analysis is applied to identify the species-specific sequences of each species. First, all possible kmers are generated from the whole genome and collected into the fragment sequence database. Kmers with PAM are retained and collected into the candidate fragment sequence database. Then, all kmers from the candidate fragment sequence database are blasted against other candidate fragment sequence databases, and kmers that exist only in the target species are loaded in the specific sequence database. In experimental operations, one of the species-specific sequences in the constructed database can be used to identify samples of unknown identity via PCR-Sanger sequencing or the CRISPR-Cas12a system.

After finding species-specific target sequences from the genome, we examined the samples for the presence of species-specific target sequences from an experimental level, using different molecular technologies (Figure 1). Here, we worked with two different technologies, Sanger sequencing and the CRISPR-Cas12a system, where the CRISPR-Cas12a system had three forms of fluorescence representation, including fluorescence values, visual fluorescence, and lateral flow tests. Sanger sequencing directly revealed sequence differences between the species, and the CRISPR-Cas12a system conducted sensitive, stable, and specific identification.

**Figure 2:**
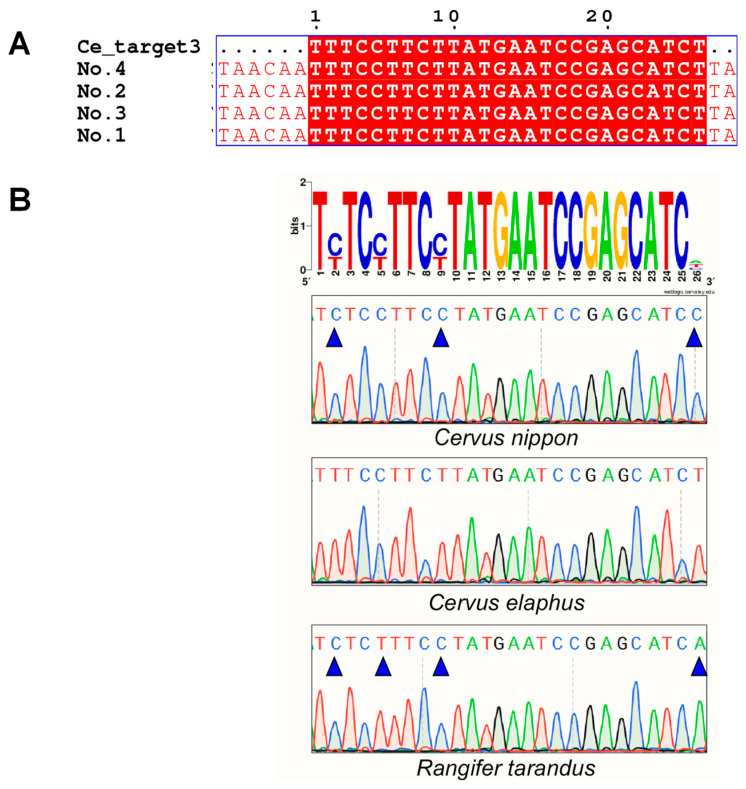
Accurate species-specific target sequence diagnostics achieved through AGE with a Sanger sequencing platform. (**A**) The alignment for the Ce_target3 sequence with the amplicons from four fresh samples of *C. elaphus*. (**B**) AGE specifically identified the specific target sequence Ce_target3 of *C. elaphus* in fresh animal samples with the Sanger sequencing platform.

For Sanger sequencing, we amplified the regions where the target sequences were located using universal primers. The results of the identification of *C. elaphus* and *C. nippon*, using Ce_target1, -2, and -3 and Cn_target1, -2, and -3, respectively, are presented in Figure 2 and Figure S1. We found that Cn_target1, -2, and -3 were present only in *C. nippon* and Ce_target1, -2, and -3 were present only in *C. elaphus*, and that there was no intraspecific variation in all target sequences. Additionally, there were different numbers of base differences between species-specific target sequences and sequences at the same position in other species. Sequence differences enabled the identification of different animal samples using AGE.

**Figure 3:**
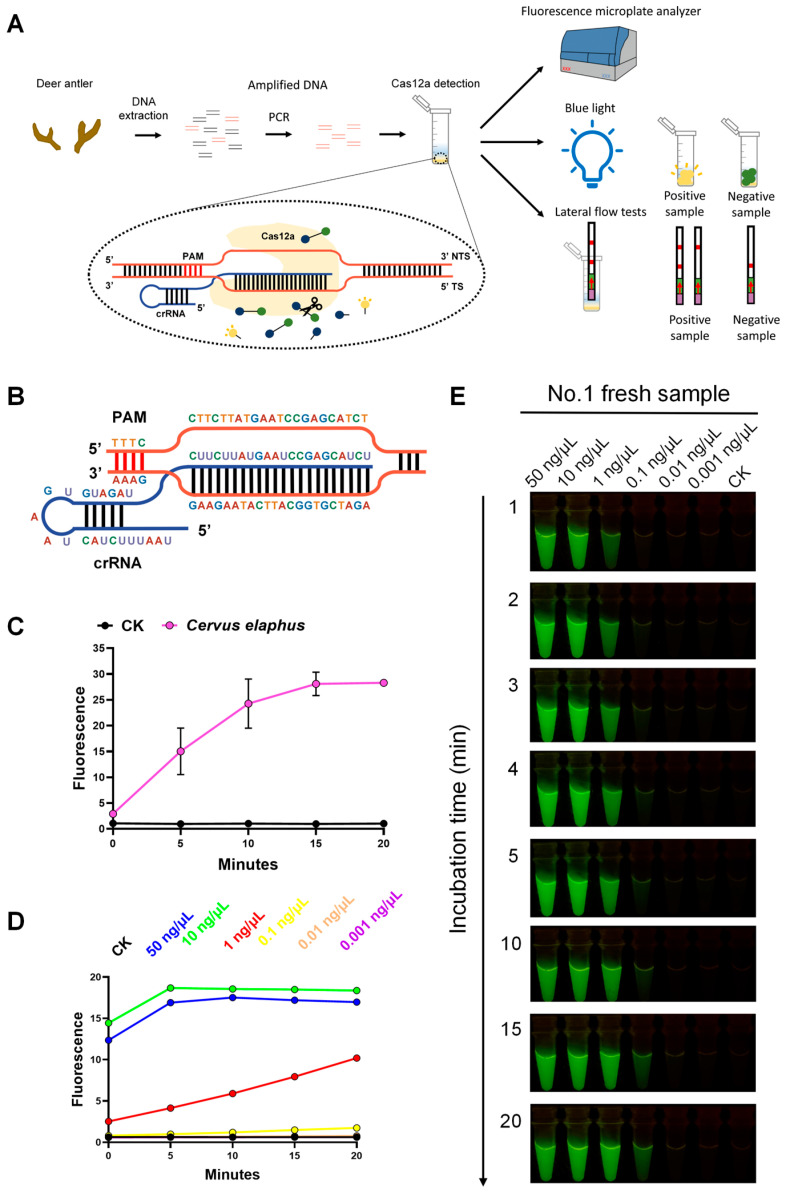
Sensitive and species-specific species identification using AGE with the CRISPR-Cas12a system. (**A**) Schematic diagram demonstrating the principles and processes of the CRISPR-Cas12a system. After extraction from samples, species-specific target sequences that bind to target-specific crRNA were amplified using PCR. Activated Cas12a subsequently generates cleavage activity that cleaves the ssDNA reporter, resulting in fluorescence. Fluorescence can be examined in three ways. (**B**) Species-specific target sequence of *C. elaphus* and its crRNA. (**C**) Specificity for the specific target sequence assay was demonstrated by fluorescence after 20 min. Each group contains all the reagents of the CRISPR system with different DNA substrates. CK: nuclease-free water. *Cervus elaphus*: purified amplicons for four *C. elaphus* fresh samples. (**D**) AGE sensitivity assay using species-specific crRNA and serial dilutions of the No.1 fresh sample-amplified DNA. Each group contains all the reagents of the CRISPR system with different DNA substrates. CK: nuclease-free water. (**E**) Sensitivity assay of visual CRISPR-Cas12a-based species identification. The reaction was examined via the blue light illuminator at 1, 2, 3, 4, 5, 10, 15, and 20 min after incubation at 37 °C. Each group contains all the reagents of the CRISPR system with different DNA substrates. CK: nuclease-free water.

As a benefit from the collateral cleavage activity of Cas12a, the progress and results of the reaction could be monitored by fluorescence. The fluorescence presentation of the CRISPR-Cas12a system contains three forms: fluorescence values, visual fluorescence, and lateral flow tests. The fluorescence values were inspected using the fluorescence microplate analyzer. We validated the feasibility, specificity, and sensitivity of the CRISPR-Cas12a system by utilizing Ce_crRNA3 as crRNA and four fresh *C. elaphus* samples as the DNA substrate. Figure 3C demonstrates the specificity of the CRISPR-Cas12a system. When the amplified fragment of the *C. elaphus* sample was present, the fluorescent microplate analyzer detected a significant fluorescence signal, which increased rapidly over a short period of time and was significantly higher than the negative control (CK). Also, the experimental reproducibility of the four *C. elaphus* samples was good. We also explored the sensitivity of the CRISPR-Cas12a system. At a DNA substrate concentration of 10 ng/μL, the sample exhibited the highest fluorescence signal and reached its maximum value in the shortest time. Additionally, even at a lower DNA substrate concentration of 1 ng/μL, the CRISPR-Cas12a system successfully found the existence of *C. elaphus*. The above results indicate that the CRISPR-Cas12a system enables sensitive, stable, and species-specific identification. 

Fluorescence can be monitored by not only the fluorescence microplate analyzer but also the blue light illuminator. The fluorescence microplate analyzer displayed the fluorescence intensity digitally, whereas the blue light illuminator made the fluorescence visible to the naked eye (Figure 3A). Compared with the instrumental measurement of fluorescence, visualized fluorescence tests were easier to realize in real time and during on-site inspections. To confirm the feasibility, sensitivity, and the most suitable observation time for the visual CRISPR-Cas12a system, we chose the No.1 fresh *C. elaphus* sample as an example and used Ce_target3 and Ce_crRNA3 as the specific target sequence and crRNA, respectively. According to Figure 3E, the fluorescence intensity in the sample reaction tube gradually increased, and the fluorescence intensity of the no-template control group was never observed with increasing incubation time. The fluorescence was significantly enhanced during the first three minutes of incubation, after which it slowed down. Taking into account the time-cost and the easily recognizable fluorescent signal, we set an incubation time of 3 min and stopped the reaction at that point. To demonstrate the sensitivity of visual AGE and discover the optimal sample DNA concentration for practical applications, the No.1 *C. elaphus* sample was tested with different concentrations. As shown in Figure 3E, there was green fluorescence when the concentrations of the sample were 50 ng/μL, 10 ng/μL, 1 ng/μL, and 0.1 ng/μL. For samples with concentrations of 0.01 ng/μL and 0.001 ng/μL, as well as for the no-template control group, there was no green fluorescence signal. The tubes of 50 ng/μL, 10 ng/μL, and 1 ng/μL showed strong fluorescence, which was almost indistinguishable to the naked eye. Considering the CRISPR-Cas12a system sensitivity results and the minimum amount of DNA used for identification, we specified the use of 10 ng/μL DNA in subsequent visual CRISPR-Cas12a system experiments.

**Figure 4:**
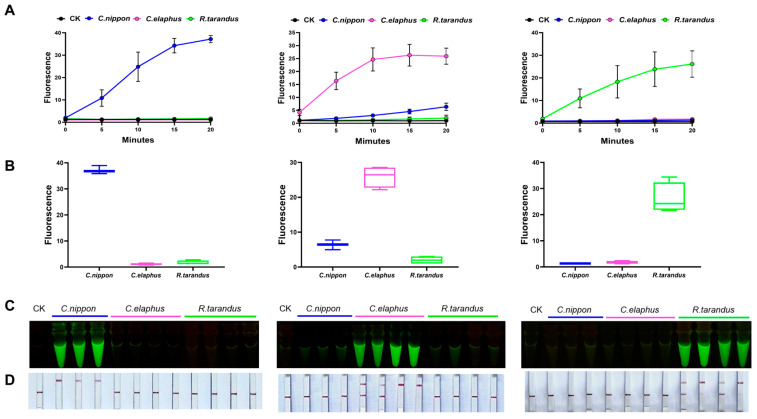
By employing the CRISPR-Cas12a system, AGE achieved the precise species identification of *C. nippon*, *C. elaphus*, and *R. tarandus* in 11 fresh animal samples. (**A**) Species-specific sequences allowed the precise identification of each target species by fluorescence. Each group contains all the reagents of the CRISPR system with different DNA substrates. CK: nuclease-free water. (**B**) Species-specificity of AGE for each sample tested by fluorescence after 20 min. (**C**) Visual AGE results showed specific identification for each target species. Each group contains all the reagents of the CRISPR system with different DNA substrates. CK: nuclease-free water. (**D**) For each species-specific assay, fresh samples were accurately identified with lateral flow. Each group contains all the reagents of the CRISPR system with different DNA substrates. CK: nuclease-free water.

Lastly, we employed the CRISPR-Cas12a system to assess the discriminatory capability of Cn_target1, -2, and -3 and Ce_target1, -2, and -3 towards *C. elaphus*, *C. nippon*, and *R. tarandus*. Remarkably, all the results of the fluorescence values, visual fluorescence, and lateral flow tests showed that only samples corresponding to the target species showed a significant fluorescent signal (Figure 4 and Figure S2), indicating that Cn_target1, -2, and -3 and Ce_target1, -2, and -3 could be specifically utilized to identify *C. nippon* and *C. elaphus*, respectively.

### 2.3. Animal Species Identification of Fresh Samples and Commercially Available Products Based on Analysis of Whole-Genome (AGE) 

Whether the research system for the target species is established or not, the application system of AGE can be divided into two cases. Once the AGE research system for the target species was established, it allowed for direct species identification using the obtained species-specific sequences, eliminating the need for further bioinformatics analysis. Similarly, if the establishment of the AGE research system for the target species has never been performed before, the analysis of whole genome of the species is required before using AGE to identify the species. We employed the CRISPR-Cas12a system with AGE to identify three related species: *C. nippon*, *C. elaphus*, and *R. tarandus*. This serves as an operational case study, showcasing the application process of AGE. The 2020 edition of “Chinese Pharmacopoeia” stipulated that deer antler velvet was made from *C. nippon* and *C. elaphus*. *R. tarandus* was the most frequent species among adulterants of this product. In light of this, we proceeded to conduct identification of 35 commercially available deer antler products and made an intriguing discovery from the results.

Having established the AGE research systems for both *C. elaphus* and *C. nippon* previously, we just selected one sequence each from the obtained three species-specific target sequences of *C. elaphus* and *C. nippon* for identification. Here, we selected the species-specific sequences Ce_target3 and Cn_target3 of *C. elaphus* and *C. nippon*, respectively, for subsequent experiments. 

However, for *R. tarandus*, the bioinformatic analysis of its genome needed to be completed in order to find its specific sequences before it could be later identified. The methods used for bioinformatics analysis of the genomic data of *R. tarandus* and the genomic analysis of *C. elaphus* were the same. Bioinformatics analysis of the genome of *R. tarandus* revealed that its fragment sequence database contained more than 2 billion sequences, of which less than 5% were candidate fragment sequences. The specific target sequence database consisted of nearly 8.5 million sequences (Table S1). Rt_target1 and Rt_target2 were chosen from the nuclear genome, while Rt_target3 was chosen from the mitochondrial genome. Also, three species-specific target sequences were also validated for sequence specificity, using the CRISPR-Cas12a system (Figure 4 and Figure S2). Rt_target3 was selected for subsequent experiments.

**Figure 5:**
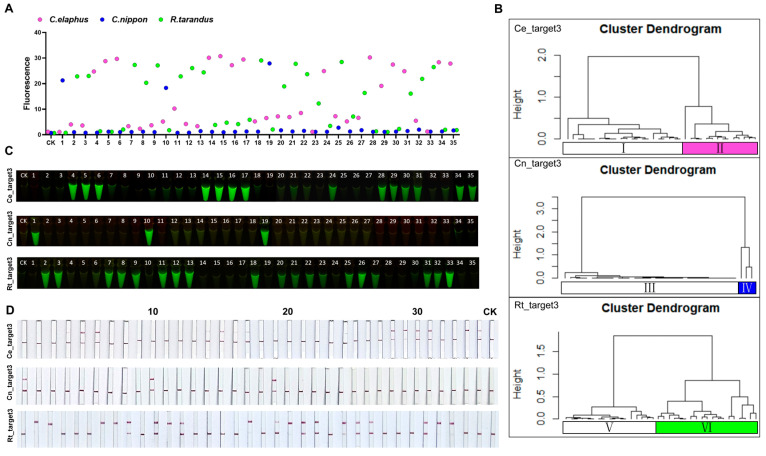
AGE successfully identified *C. nippon*, *C. elaphus*, and *R. tarandus* in 35 commercially available products using the CRISRP-Cas12a system. (**A**) Species identification for 35 commercially available products tested by fluorescence after 20 min. Each group contains all the reagents of the CRISPR system with different DNA substrates. CK: nuclease-free water. (**B**) Cluster analysis results of 35 commercially available products on the fluorescence values of three species-specific target sequences at 20 min. Samples whose fluorescence values cluster with the CK group are considered to not contain the target species, while those that do not cluster with the CK group are regarded as containing the target species in the sample. I contains 1, 2, 3, 7, 8, 9, 10, 11, 12, 13, 18, 19, 20, 21, 22, 23, 25, 26, 27, 32, 33, and CK; II contains 4, 5, 6, 14, 15, 16, 17, 24, 28, 29, 30, 31, 34, and 35; III contains 2, 3, 4, 5, 6, 7, 8, 9, 11, 12, 13, 14, 15, 16, 17, 18, 20, 21, 22, 23, 24, 25, 26, 27, 28, 29, 30, 31, 32, 33, 34, 35, and CK; IV contains 1, 10, and 19; V contains 1, 4, 5, 6, 10, 14, 15, 16, 17, 19, 24, 28, 29, 30, 34, 35, and CK; VI contains 2, 3, 7, 8, 9, 11, 12, 13, 18, 20, 21, 22, 23, 25, 26, 27, 31, 32, and 33. (**C**) Species identification for 35 commercially available products tested by the visual CRISPR-Cas12a system. Each group contains all the reagents of the CRISPR system with different DNA substrates. CK: nuclease-free water. (**D**) Species identification for 35 commercially available products tested by lateral flow. Each group contains all the reagents of the CRISPR system with different DNA substrates. CK: nuclease-free water.

All the DNA samples extracted from the 35 antler commodities were successfully amplified using cytochrome C oxidase subunit I (COI) universal primers and sequenced. Table S3 presents the BLAST results for the 35 commercially available products. Using DNA extracted from samples, we assayed 35 antler commodities using three species-specific crRNAs, which stood for *C. elaphus*, *C. nippon*, and *R. tarandus*. In 13 samples, *C. elaphus* was exclusively detected, while in three samples, *C. nippon* was exclusively detected, and, in 18 samples, *R. tarandus* was exclusively detected. Also, the No.31 sample was found to be a mixture of *C. elaphus* and *R. tarandus*, which was in agreement with the preliminary results of DNA barcoding (Figure 5).

### 2.4. Generating Species Identification from Mixtures 

Based on sequencing peak analysis and the results of AGE, sample No. 31, from Changchun, Jilin Province, was preliminarily identified as a mixture. Subsequently, we proceeded to investigate its identification through molecular cloning. Twenty clones were selected, i.e., 20 COI sequences were sequenced for analysis. Out of the twenty COI sequences obtained from the No.31 sample, eight were identified as *Przewalskium albirostris* sequences, two as *C. elaphus* sequences, six as *R. tarandus* sequences, and three as moths and flying insect sequences; one sequence did not match the closest species, indicating that this sample was a mixture. Eight COI clone sequences of *P. albirostris* were categorized into two distinct haplotypes, two of *C. elaphus* were classified into two different haplotypes, and six of *R. tarandus* were classified into three diverse haplotypes.

**Figure 6:**
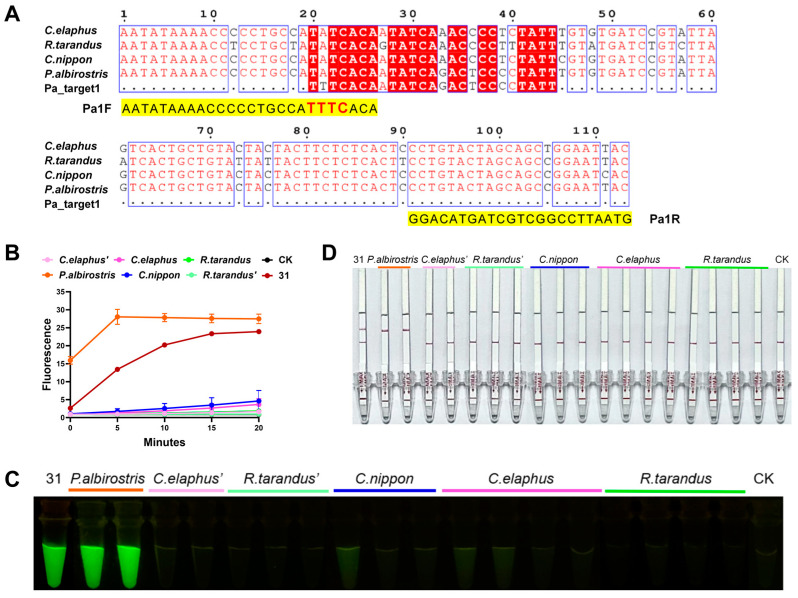
The application of AGE in mixtures. (**A**) The process of adding the PAM structure to the PAM-free species-specific target sequences (**B**) Specific identification of *P. albirostris* for the mixture, monoclonal samples, and fresh samples tested by fluorescence. Each group contains all the reagents of the CRISPR system with different DNA substrates. CK: nuclease-free water; 31: No.31 commercially available product. (**C**) Specific identification of *P. albirostris* for the mixture, monoclonal samples, and fresh samples tested by visual fluorescence. Each group contains all the reagents of the CRISPR system with different DNA substrates. CK: nuclease-free water; 31: No.31 commercially available product. (**D**) Specific identification of *P. albirostris* for the mixture, monoclonal samples, and fresh samples tested by lateral flow. Each group contains all the reagents of the CRISPR system with different DNA substrates. CK: nuclease-free water; 31: No.31 commercially available product.

In addition to *C. elaphus* and *R. tarandus*, the mixture was dominated by *P. albirostris*. This intriguing finding warrants further in-depth exploration and investigation. To validate the broad applicability of AGE, we employed it to conduct an identification of *P. albirostris* identification as well. We intended to derive the specific sequence of *P. albirostris* from the cloned COI sequence and the COI sequences of 11 fresh animal samples. Since only PAM-free *P. albirostris*-specific sequences were found, located within the COI sequences, we added the PAM structure to the sequence (Pa_target1) by designing amplification primers. Finally, AGE was performed, with Pa_target1 as the target, and the matched crRNA (Pa_crRNA1) was synthesized based on this. The COI fragments of 11 fresh animal samples and all the obtained single-haplotype DNA samples (two *P. albirostris* clone DNA samples, two *C. elaphus* clone DNA samples, and three *R. tarandus* clone DNA samples) were used as DNA substrates for their respective assays. As we can see in Figure 6, only the samples containing *P. albirostris* showed significant fluorescence, which shows that AGE had great specificity for different species.

## 3. Discussion

### 3.1. Analysis of Whole-Genome (AGE) Identification System Developed via Whole Genome Analysis in the Animal Kingdom

We analyzed genome sequences from a species-specific perspective. Previous studies on genomes have mainly focused on two aspects. The first studies the functions of genes in the genome, revealing the relationship between genes and phenotypes and the mechanism of gene action in organisms [22]. The second investigates the association between genomes and diseases, including the genetic risk factors for diseases and the discovery and application of genetic markers. We exploited gene sequence differences in whole genomes of different species to achieve species identification. Based on this, we have devised an identification method called AGE and established the AGE identification system for the animal kingdom. 

This study establishes the feasibility of developing an identification method based on AGE for animals. The core innovation of this study lies in the identification of species through whole genome analysis, wherein species-specific sequences are extracted from the whole genome, including both nuclear and mitochondrial genomes. The vast majority of studies have used the COI gene to identify animals such as mammals [23], snakes [24], fish [25,26,27], and insects [28,29,30,31]. Some studies have also used four additional DNA fragments, cytochrome b (cyt b), 16S rDNA, ITS2, and 12S rDNA for animal identification. The DNA fragment sequences identified for species identification are highly conserved, and there have been minimal developments of new identification sequences. In contrast to previous identifications, which primarily relied on DNA barcodes or commonly accepted sequences for species identification, AGE begins with the whole genome to pinpoint target species. From the genomes of *C. elaphus*, *C. nippon*, and *R. tarandus*, we randomly selected two species-specific target sequences located in the nuclear genome and one species-specific target sequence located in the mitochondrial genome for each species. All nine species-specific target sequences effectively identified their respective target species within animal samples; additionally, three species-specific target sequences selected from the nine were also successful in identifying their corresponding target species within commercially available products containing animal ingredients. Of the nine involved in the paper, six species-specific sequences from the nuclear genome did not have any annotation information. These totally new sequences were called “dark matter” in the genome. In addition to previously unstudied sequences, we also found target sequences that had annotation information but were not taken up for identification. Cn_target3 is located in the D-Loop region of mitochondrial DNA, and Ce_target3 is located in the gene-encoding NADH dehydrogenase subunit 1(ND1). Studies on the D-loop have centered around its involvement in telomere maintenance [32], DNA damage repair [33], and disease processes [34,35,36], while research on ND1 has primarily focused on its role in various cancer processes [35,37]. Apart from the aforementioned cases, we also found species-specific target sequences, including Rt_target3 located in the COI sequence, within common sequences utilized for identification purposes. The fact that species-specific target sequences can be identified from universal barcode sequences used in animal identification indicates the effectiveness of the AGE bioinformatics analysis workflow. 

Three species-specific sequences from the mitochondrial genome were located in the gene region, while the remaining six species-specific sequences from the nuclear genome were located in the intergenic region. Species-specific sequences can be present in either the gene region, the intergenic region, or they can extend across both regions. The majority of previous studies on the genome have been focused on sequences within gene regions and intergenic regions that impact gene expression. AGE discards the preference of previous studies for the aforementioned genomic sequences, adopts species specificity as the sole selection criterion, and finds “dark matter” and other specific sequences in the genome. From the perspective of species identification, AGE effectively explores the information within the genome and pioneers the identification potential of functional genes and unknown sequences in the genome. Furthermore, the species-specific target sequences selected by AGE showed significant efficacy in species identification. In summary, we have successfully applied a whole genome analysis strategy to species identification in the animal kingdom and developed a stable and specific method for animal species identification, AGE.

### 3.2. Species Identification via Analysis of Whole-Genome (AGE) in Two Layers: Research and Application

AGE enables species identification in two layers: research and application. The establishment of the AGE research system consists of two steps: first, the identification of species-specific target sequences from the whole genome using bioinformatics technologies at the data layer; and second, the identification of species-specific target sequences in samples, using different molecular technologies at the experimental layer in order to achieve species identification. Once the AGE research system for a species is successfully established, the species can be identified by AGE without bioinformatics analysis, and the appropriate specific target can be selected directly from the database of specific target sequences established at the research layer for the experiment. Therefore, the AGE application system consists of just one step: recognizing species-specific sequences using different experimental technologies. As shown in the testing of commercially available products using AGE, we chose just one sequence from the three validated species-specific target sequences for identifying *C. elaphus*, and, likewise, one sequence for identifying *C. nippon*. Given that the AGE research system for *R. tarandus* had not yet been established, before testing for the presence of *R. tarandus* in the sample, we needed to perform a bioinformatics analysis on the *R. tarandus* genome to generate a *R. tarandus*-specific target sequence database. From this database, we randomly picked three species-specific target sequences to identify the fresh animal samples from *R. tarandus*. Subsequently, we selected one validated species-specific target sequence from the three to test for the presence of *R. tarandus* in commercially available products.

The species-specific target sequence databases eliminate preliminary basic work, such as collecting and analyzing genomes, reduce the effort and time spent on bioinformatics analysis, and largely simplify the operation of AGE. A real-time online database can help researchers avoid redundant work and prevent the wastage of significant human and material resources. We propose to create an AGE online database website, containing all eukaryotic species-specific target sequences for reference use by others. The AGE online database site will provide all the information needed for the sample identification process by AGE, including a database of species-specific target sequences, amplification primer sequences, instrumentation reagents, operational procedures, etc. This study has constructed the specific target sequence databases of *C. elaphus*, *C. nippon*, and *R. tarandus*, providing examples for the construction of specific target sequence databases for other eukaryotic species. In addition, we have successfully identified animal samples of the endangered species *C. nippon*, as well as commercially available products whose components include *C. nippon*, using sequences from the *C. nippon*-specific target sequence database. This database not only simplifies the steps of AGE bioinformatics analysis but also holds potential application value in various fields such as inspection and quarantine as well as market surveillance. 

### 3.3. Compatibility of Analysis of Whole-Genome (AGE) with Multiple Molecular Technologies Enhances Its Versatility

AGE can utilize various molecular technologies as implementation tools for species identification. In this study, we used Sanger sequencing technology and the CRISPR-Cas12a system to inspect species-specific target sequences. Different molecular technologies have their own characteristics. Sanger sequencing technology provides direct base differences in sequences between different species. The CRISPR-Cas12a system provides multiple presentations of identification results, such as fluorescence values, visual fluorescence, and lateral flow tests. Quantification of the identification results is achieved through fluorescence values, which provide richer information. Visual fluorescence and lateral flow tests do not require instruments, and the results are visible to the naked eye, making them more intuitive. Moreover, the CRISPR-Cas12a system can combine DNA fast extraction technology with room temperature amplification technology to present results with visual fluorescence or lateral flow tests. It has strong portability and the potential to enable on-site identification. The reaction system can be packaged and developed as a commercial kit, with strong commercial value. In addition to the two molecular technologies mentioned in the paper, we believe that, by modifying the sequence selection criteria in bioinformatics analysis, AGE can be combined with various other molecular technologies, such as simple PCR, quantitative real-time PCR (qPCR), and more. In the post-pandemic era of COVID-19, qPCR technology has been extensively adopted and offers advantages such as the widespread availability of instruments and the widespread proficiency of operators. Different molecular technologies have varying economic costs and sensitivities. Given that AGE can be combined with multiple molecular technologies for identification, individuals can consider the sensitivity requirements and economic costs of the assay and choose the most suitable molecular technique for their experiments. AGE can meet different needs in different scenarios during the appraisal process and has wide applicability.

### 3.4. Analysis of Whole-Genome (AGE) Performs Well When Identifying Mixed DNA Samples

In the analysis of species in commercially available products, we had an interesting and confusing discovery. Heterozygosity was present in the sequencing chromatogram of COI sequences of the No.31 sample. The COI sequence of the sample aligned with *R. tarandus* in the NCBI database. Using the CRISPR-Cas12a system, the AGE identification results for the sample indicated the presence of *C. elaphus* and *R. tarandus*. To confirm the presence of *C. elaphus* and *R. tarandus* in the sample, we conducted further identification research using molecular cloning methods. The results of the molecular cloning experiments showed that the samples contained not only the Cervidae family, such as *C. elaphus*, *R. tarandus,* and *P. albirostris*, but also small flying insects, such as *Lachesilla pedicularia* and *Tineola bisselliella*. The results of the comparison were that eight of the sixteen COI sequences of the *Cervidae* family were matched to *P. albirostris*, six to *R. tarandus,* and two to *C. elaphus*, indicating that most of the components in this sample were *P. albirostris*, followed by *R. tarandus*, and then a few that were *C. elaphus*. Through the obtained COI sequence, we also identified the specific target sequence for *P. albirostris*. Using AGE, we successfully identified the presence of *P. albirostris* in the mixed sample and the DNA sample obtained through cloning, which contained a *P. albirostris* COI sequence. AGE’s simplicity and efficiency in solving the complex challenges of mixture identification highlight its effectiveness. AGE is a very suitable method when one wants to determine whether a sample is a mixture of certain species.

### 3.5. Factors That May Affect Species Identification by Analysis of Whole-Genome (AGE)

Whole genome analysis is the basis for animal species identification via AGE. The key to the successful implementation of AGE is the collection of genomes. When the genome of the target species is publicly available, genomic data can be obtained by contacting the authors of publications or downloading them from websites. However, for the 8.7 million or so eukaryotic species predicted by biologists to be present on Earth, only a very small fraction have publicly available genomes. To address this issue, we recommend using shallow sequencing technology to obtain species’ genomes. Studies have shown that the shallow sequencing of species can effectively provide genomic information [38]. In fact, the example of *C. nippon* mentioned earlier demonstrates that species’ genomes, obtained through shallow sequencing techniques, can fulfill the genomic requirements for bioinformatic analysis using AGE. Due to the rapid development of sequencing technology, the cost of genome sequencing has fallen dramatically in recent years. According to the National Human Genome Research Institute (NHGRI), the cost of sequencing a 1 Mb genome in 2021 was only USD0.006. According to NCBI, 95% of animal genomes are between 1 Mb and 3 Gb in size. Therefore, it is now typical to obtain a whole genome of an animal species using 30× shallow sequencing for less than USD540 or as little as USD0.18. The largest animal genome currently known is *Euphausia superba*, which is 48.01 Gb in size [39], and the cost of obtaining its 30 × genomic data is USD8641.8, which is also within the range of research funding. Thus, the acquisition of genomes should not be a limiting factor for the development of AGE, which requires a lot of financial and human resources for the establishment of the preliminary research system and database construction. As with the Human Genome Project, the establishment of the preliminary research system for AGE and the construction of databases also require a massive amount of money and manpower. Establishing all eukaryotic species-specific target sequences is a distant goal that requires the concerted efforts of scientists worldwide. Still, the use of databases significantly streamlines AGE and holds immense potential for practical applications. This process could attract multidisciplinary talent and garner increased financial support for the field of identification. Like other DNA molecular identification techniques, AGE is unable to identify samples that do not contain DNA or samples with severely degraded DNA.

### 3.6. Potential of Analysis of Whole-Genome (AGE) to Tackle Identification Challenges

To ensure the correctness of the materials used and the reliability of the established AGE system, we chose *C. elaphus* and *C. nippon*, which can be distinguished by universal barcodes, as model species. AGE is highly accurate as it utilizes the principle that genome sequences of different species must differ to carry out species identification. Just as there are no two identical leaves in the world, there are inevitable differences between species at the genomic level. By analyzing the genome of one species against the genomes of other species, it is possible to find species-specific target sequences that could identify that species. Thus, AGE has the potential to identify any two species, especially for closely related species that are difficult to distinguish, which have garnered significant attention, for example, ants [40,41,42], butterflies [43,44], bees [45], spiders [46,47], and fish [48].

### 3.7. Analysis of Whole-Genome (AGE) Helps Protect Biodiversity

Illegal hunting and the trade of wildlife are rampant. The wildlife trade involves thousands of species in the wild, including more than 7600 or nearly a quarter of all terrestrial vertebrate species, and is worth billions of dollars annually [49]. In order to protect endangered species, countries have signed the Convention on International Trade in Endangered Species of Wild Fauna and Flora (CITES). To enable the member countries of CITES to successfully prosecute illegal trade cases, reliable species identification methods are needed [50]. This study utilizes shallow sequencing technology to obtain the genome of *C. nippon* and relies on AGE to successfully identify *C. nippon* animal samples as well as commercial products containing *C. nippon* ingredients. *C. nippon* is classified as a Class I protected species in China and is listed in the International Union for Conservation of Nature (IUCN) Red List of Threatened Species in 2015. AGE serves as a powerful tool for monitoring the trade of endangered species. Additionally, AGE can be applied as an effective method for the strict prevention and control of invasive species. Accurate species identification by AGE could help strengthen the prevention and control of exotic species ports, strictly prevent the introduction of other exotic species, and build a solid port quarantine defense.

### 3.8. Analysis of Whole-Genome (AGE) as a Powerful Tool for the Regulation of Commercial Products

The market is flooded with counterfeit and misrepresented products due to confusion when identifying species, and the mislabeling of commercial products is also common. This situation raises significant concerns regarding food and drug safety. AGE has the potential to provide information about species’ compositions and the honesty of ingredient declarations. In this study, DNA was extracted from 35 antler samples purchased nationwide and molecularly identified using DNA barcoding and AGE identification systems. The results revealed that the commercial deer antler samples originated from *C. elaphus*, *C. nippon*, and *R. tarandus*. The identification results from both DNA barcoding and AGE were consistent, indicating a complex origin of the commercial deer antler samples; they were primarily sourced from *R. tarandus* and contained mixtures of *C. elaphus* and *R. tarandus*, as well as impurities. The investigation of product labels showed that 26 out of the 35 commercial products were labeled as “deer antler” without specifying the composition, while the remaining nine products were labeled as “sika deer antler” or “red deer antler”. The identification results revealed that the actual ingredients of five of the commercially available products did not match the labels, including the substitution of *C. elaphus* for *C. nippon* or *R. tarandus* for *C. elaphus* and *C. nippon*. It is evident that discrepancies between product labels and actual test results, as well as product adulteration, are common in the tested samples. It is no coincidence that product quality issues were also found in commercially available herbal products [51], fish products [52], and in the food service industry [53]. To safeguard consumer rights and avoid the risk of violating certain religious and/or cultural strictures, AGE provides accurate identification of species and enables effective control of the production and distribution of goods.

## 4. Materials and Methods

### 4.1. Materials’ Preparation

A total of 46 antler samples were collected, including 11 fresh animal samples and 35 commercially available antler products. Eleven fresh samples consisted of four *C. elaphus* samples, three *C. nippon* samples, and four *R. tarandus* samples, which were stored at −80 °C. Fresh samples for the establishment of the AGE research system were identified by Professor Chunyi Li and Hengxing Ba from the Institute of Special Animal and Plant Sciences, Chinese Academy of Agricultural Sciences. All fresh samples were purchased from farms. These farms have obtained the “National Key Protected Animal Breeding License”, ensuring that all purchases were legal and compliant. In addition, 35 samples of commercially available antlers were collected from pharmacies, herbal medicine companies, and e-commerce platforms, including 19 antler powders, 14 antler slices, and two antlers. All samples were sequenced, and their origin was identified by alignment with the COI sequences contained in the National Center for Biotechnology Information (NCBI) and an online DNA barcoding database for herbal materials [54] (http://www.tcmbarcode.cn/china/, accessed on 3 February 2023) using the BLAST program. The experimental materials and their species identification results are detailed in Tables S3 and S6.

### 4.2. Bioinformatics Analysis of Target Species’ Genomes 

The genomes of *C. elaphus* (GCF_910594005.1, NC_007704.2) and *R. tarandus* (GCA_004026565.1, NC_007703.1) were downloaded from the NCBI database (https://www.ncbi.nlm.nih.gov, accessed on 30 December 2021). The genome of *C. nippon* (GWHBJVV00000000) was downloaded from the NGDC database (https://ngdc.cncb.ac.cn/biocode, accessed on 3 February 2023). For *C. elaphus* and *R. tarandus*, the genomes (L = genome length) were cut into K bp fragments using Jellyfish (v1.1.12) [55] to generate (L-K + 1) kmers with the copy number using the default parameters (K: 23–27) [56]. The kmers with PAM (TTTV starting or VAAA ending) sequences were extracted and compared to their genomes, respectively, using Bowtie (v1.1.0) [57] with the default parameters to locate their position in the genome. The kmers that have a PAM sequence and map perfectly to the genome of the target species were named “the candidate fragment sequences”. Then, the candidate fragment sequences of one species were mapped to “the candidate fragment sequence” of two other species using Bowtie (v1.1.0) [57]. Only candidate fragment sequences with sequence differences of three or more mismatches and all indels were retained, and these were called “the specific target sequences”. For *C. nippon*, 120 Gb of sWGS data (~50×) was bioinformatically analyzed in the same way as that of the genome of *C. elaphus*. The specificity of “the specific target sequences” was analyzed by mapping them to the genomes of other species, using Cas-OFFinder (v2.4) [58].

The crRNAs were designed based on the selected specific target sequences according to the manufacturer’s instructions and were synthesized and purified by GenScript.

### 4.3. DNA Extraction, Polymerase Chain Reaction (PCR) Amplification, and Purification 

Fresh samples were ground in liquid nitrogen in powder form. Genomic DNA extraction was conducted on sample powders using the TIANamp Genomic DNA Kit (DP304, Tiangen Biotech (Beijing) Co., Ltd., Beijing, China). We have made some adjustments in accordance with the manufacturer’s protocol. The sample was incubated at 56 °C overnight in Buffer A and Proteinase K solution. DNA concentration and quality were estimated using a NanoDrop 2000C UV evispectrophotometer (Thermo Fisher Scientific Inc., Beijing, China), and the integrity of the DNA was tested by agarose gel electrophoresis. 

The primers and reaction conditions used for amplification are listed in Table S2. Each 50 μL PCR mixture contained 300 ng of genomic DNA, 25 μL of 2× Taq PCR MasterMix (Aidlab Biotechnologies Co., Ltd., Beijing, China) and 1 μL each of the forward and reverse primers (10 μmol/L). The PCR products were examined via electrophoresis in a 2% agarose gel. After amplification, PCR products were purified using the Universal DNA Purification Kit (DP214, Tiangen Biotech (Beijing) Co., Ltd., Beijing, China), following the manufacturer instructions, and they were assessed using a NanoDrop 2000C UV evispectrophotometer (Thermo Fisher Scientific Inc., China).

### 4.4. Species-Specific Target Sequence Acquisition

The bidirectional sequencing of purified PCR products was undertaken using Sanger sequencing. Contig assembly and consensus sequence generation were performed using CodonCode Aligner.

### 4.5. CRISPR-Cas12a System 

The CRISPR-Cas12a system was performed in a 100 μL volume containing 10 μL 10× NEB buffer 2.1 (New England Biolabs (Beijing) Ltd., Beijing, China), 2 μL Cas12a (final concentration: 33 nM), 3.3 μL crRNA (final concentration: 300 nM), 10 μL substrate DNA, 4 μL ssDNA (/5′6-FAM/CCCCCCCCCC/3′BHQ-1, final concentration: 400 nM), and 70.7 μL nuclease-free water. Cas12a and crRNA were incubated at 37 °C for 10 min in 1× NEBuffer. Then, the substrate DNA and ssDNA were added. The reaction mixture was incubated at 37 °C and the fluorescence intensity was measured at λ ex 483 nm/λ em 535 nm using a fluorescence microplate analyzer (Thermo Fisher Co., Ltd., Waltham, MA, USA). The fluorescence intensity also could be inspected using a blue light illuminator (BG-Vtrans520s, Baygene Biotech (Beijing) Co., Ltd., Beijing, China). After being incubated at 37 °C for 10 min, the test strips were placed in the reaction mixture. The results of the fluorescence were observed and saved using a smartphone.

Furthermore, Cas12a reactions were detected using commercially available lateral flow strips (TS104, Suzhou Gendx Biotech Co., Ltd., Suzhou, China). To enable lateral flow visualization, a custom FAM-Biotin reporter (/5′6-FAM/TTATTATT/3′Bio) was incorporated at a final concentration of 40 nM. The 100 µL reactions were incubated at 37 °C for 10 min, followed by the insertion of lateral flow strips into the tubes containing the reactions. After incubating at room temperature for 5 min, images were captured using a smartphone.

## 5. Conclusions

In this study, we have successfully established an Analysis of whole-GEnome (AGE) identification system in the animal field. AGE achieves species identification by identifying species-specific sequences from both bioinformatics and experimental layers. Specific sequences identified through bioinformatics analysis, including those from the genomic “dark matter” (sequences without any annotation information), sequences annotated but not previously used for identification, and common DNA barcodes, have been shown to possess species identification capabilities. Then, AGE’s stable identification of the rare species *Cervus nippon* and its closely related species, *Cervus elaphus* and *Rangifer tarandus*, in animal samples was emphasized in this study. In addition, AGE identified 35 commercial products, revealing their complex origins, and a mixture was found among them, which proves the accuracy and potential wide applications of AGE. Furthermore, the establishment of the AGE identification system for *C. nippon* demonstrates the feasibility of using AGE to identify species with undisclosed genomes. By comparing species identification with and without the AGE research system, we have shown that the establishment of the AGE research system significantly simplifies the operations in AGE applications. In summary, with its precision and applicability, AGE emerges as a powerful tool for addressing the challenges of animal species identification, providing crucial support for biodiversity conservation and commercial product regulation.

## Figures and Tables

**Table 1:** Specific targets for the identification of *C. elaphus*, *C. nippon*, and *R. tarandus*.

Species	Sequence Location	No.	Specific Target Sequence (5′→3′)	Annotation	CrRNA (5′→3′)
*Cervus* *elaphus*	chromosome 29	Ce_target1	TTTGGGGAACTTAAGACTTGGGCAT	unannotated	UAAUUUCUACUAAGUGUAGAU GGGAACUUAAGACUUGGGCAU
	chromosome 29	Ce_target2	TTTGGGTCTAGATACTCATCTTCCG	unannotated	UAAUUUCUACUAAGUGUAGAU GGTCTAGATACTCATCTTCCG
	mitochondrial genome	Ce_target3	TTTCCTTCTTATGAATCCGAGCATCT	ND1	UAAUUUCUACUAAGUGUAGAU CUUCUUAUGAAUCCGAGCAUCU
*Cervus nippon*	nuclear genome	Cn_target1	TTTGAATCTGGACGGACATCCAGCC	unannotated	UAAUUUCUACUAAGUGUAGAU AAUCUGGACGGACAUCCAGCC
	nuclear genome	Cn_target2	TTTGACACCGGTCTACTGGCCTGCC	unannotated	UAAUUUCUACUAAGUGUAGAU ACACCGGUCUACUGGCCUGCC
	mitochondrial genome	Cn_target3	TTTATGTACCATTGTACATGTGTGC	D-loop	UAAUUUCUACUAAGUGUAGAU UGUACCAUUGUACAUGUGUGC
*Rangifer tarandus*	chromosome 29	Rt_target1	TTTCGTTACCCCTCCGTCGTCGGGA	unannotated	UAAUUUCUACUAAGUGUAGAU GUUACCCCUCCGUCGUCGGGA
	chromosome12/13	Rt_target2	TTTGAGATTGCCAATGTCGCGGTCG	unannotated	UAAUUUCUACUAAGUGUAGAU AGAUUGCCAAUGUCGCGGUCG
	mitochondrial genome	Rt_target3	TTTCTACTTCTTCTAGCATCATCCA	COI	UAAUUUCUACUAAGUGUAGAU UACUUCUUCUAGCAUCAUCCA

## Data Availability

Data are available within the article or its Supplementary Materials.

## Supplementary Materials

The following supporting information can be downloaded at: https://www.mdpi.com/article/10.3390/ijms25052955/s1.

## References

[1] Dirzo R., Young H.S., Galetti M., Ceballos G., Isaac N.J.B., Collen B. (2014). Defaunation in the Anthropocene. Science.

[2] Mueller U.G., Wolfenbarger L.L. (1999). AFLP genotyping and fingerprinting. Trends Ecol. Evol..

[3] Wolf C., Burgener M., Hübner P., Lüthy J. (2000). PCR-RFLP Analysis of Mitochondrial DNA: Differentiation of Fish Species. LWT-Food Sci. Technol..

[4] Hai X., Liu G.-Q., Luo J.-X., Guo Y.-S., Qian J.-P., Ya M., Guo L. (2020). Triplex real-time PCR assay for the authentication of camel-derived dairy and meat products. J. Dairy Sci..

[5] Hebert P.D.N., Cywinska A., Ball S.L., deWaard J.R. (2003). Biological identifications through DNA barcodes. Proc. Biol. Sci..

[6] Chen S., Yin X., Han J., Sun W., Yao H., Song J., Li X. (2023). DNA barcoding in herbal medicine: Retrospective and prospective. J. Pharm. Anal..

[7] Xin T., Xu Z., Jia J., Leon C., Hu S., Lin Y., Ragupathy S., Song J., Newmaster S.G. (2018). Biomonitoring for traditional herbal medicinal products using DNA metabarcoding and single molecule, real-time sequencing. Acta Pharm. Sin. B.

[8] Jia J., Xu Z., Xin T., Shi L., Song J. (2017). Quality Control of the Traditional Patent Medicine Yimu Wan Based on SMRT Sequencing and DNA Barcoding. Front. Plant Sci..

[9] Chen X., Xiang L., Shi L., Li G., Yao H., Han J., Lin Y., Song J., Chen S. (2017). Identification of crude drugs in the Japanese pharmacopoeia using a DNA barcoding system. Sci. Rep..

[10] Hao L., Xu W., Qi G., Xin T., Xu Z., Lei H., Song J. (2022). GAGE is a method for identification of plant species based on whole genome analysis and genome editing. Commun. Biol..

[11] Gan Y., Xin T., Xu W., Hao L., Qi G., Lou Q., Song J. (2023). Principles and strategies for species identification based on analysis of whole-genome. Acta Pharm. Sin..

[12] Qi G., Hao L., Gan Y., Xin T., Lou Q., Xu W., Song J. (2024). Identification of closely related species in Aspergillus through Analysis of Whole-Genome. Front. Microbiol..

[13] Sanger F., Air G.M., Barrell B.G., Brown N.L., Coulson A.R., Fiddes J.C., Hutchison C.A., Slocombe P.M., Smith M. (1977). Nucleotide sequence of bacteriophage φX174 DNA. Nature.

[14] Gao L., Xu W., Xin T., Song J. (2023). Application of third-generation sequencing to herbal genomics. Front. Plant Sci..

[15] Lewin H.A., Robinson G.E., Kress W.J., Baker W.J., Coddington J., Crandall K.A., Durbin R., Edwards S.V., Forest F., Gilbert M.T.P. (2018). Earth BioGenome Project: Sequencing life for the future of life. Proc. Natl. Acad. Sci. USA.

[16] Mora C., Tittensor D.P., Adl S., Simpson A.G.B., Worm B. (2011). How Many Species Are There on Earth and in the Ocean?. PLoS Biol..

[17] Bunn H.T. (1981). Archaeological evidence for meat-eating by Plio-Pleistocene hominids from Koobi Fora and Olduvai Gorge. Nature.

[18] Thomas S. (2006). Animal research and the search for understanding. Nat. Genet..

[19] Ma T., Tao J., Yang M., He C., Tian X., Zhang X., Zhang J., Deng S., Feng J., Zhang Z. (2017). An AANAT/ASMT transgenic animal model constructed with CRISPR/Cas9 system serving as the mammary gland bioreactor to produce melatonin-enrich milk in sheep. J. Pineal Res..

[20] Han R., Han L., Zhao X., Wang Q., Xia Y., Li H. (2023). Haplotype-resolved Genome of Sika Deer Reveals Allele-specific Gene Expression and Chromosome Evolution. Genom. Proteom. Bioinform..

[21] Xing X., Ai C., Wang T., Li Y., Liu H., Hu P., Wang G., Liu H., Wang H., Zhang R. (2023). The First High-quality Reference Genome of Sika Deer Provides Insights into High-tannin Adaptation. Genom. Proteom. Bioinform..

[22] De La Peña R., Hodgson H., Liu J.C.-T., Stephenson M.J., Martin A.C., Owen C., Harkess A., Leebens-Mack J., Jimenez L.E., Osbourn A. (2023). Complex scaffold remodeling in plant triterpene biosynthesis. Science.

[23] Tobe S.S., Kitchener A.C., Linacre A.M.T. (2010). Reconstructing Mammalian Phylogenies: A Detailed Comparison of the Cytochrome b and Cytochrome Oxidase Subunit I Mitochondrial Genes. PLoS ONE.

[24] Dubey B., Meganathan P.R., Haque I. (2011). DNA mini-barcoding: An approach for forensic identification of some endangered Indian snake species. Forensic Sci. Int. Genet..

[25] Nascimento M.H.S., Aragão D.G., Silva J.L.N., Lima R.C., Birindelli J.L.O., Fraga E.C., Barros M.C. (2023). The DNA barcode reveals cryptic diversity and a new record for the genus *Leporinus* (Characiformes, Anostomidae) in the hydrographic basins of central northern Brazil. PeerJ.

[26] Da Silva De Souza C., Mattox G., Vita G., Orrego L., Melo B.F., Oliveira C.D. (2023). Molecular species delimitation and description of a new species of *Phenacogaster* (Teleostei, Characidae) from the southern Amazon basin. ZooKeys.

[27] Ward R.D., Zemlak T.S., Innes B.H., Last P.R., Hebert P.D.N. (2005). DNA barcoding Australia’s fish species. Philos. Trans. R. Soc. B Biol. Sci..

[28] Armstrong K.F., Ball S.L. (2005). DNA barcodes for biosecurity: Invasive species identification. Philos. Trans. R. Soc. B Biol. Sci..

[29] Lv J., Wu S., Zhang Y., Chen Y., Feng C., Yuan X., Jia G., Deng J., Wang C., Wang Q. (2014). Assessment of four DNA fragments (COI, 16S rDNA, ITS2, 12S rDNA) for species identification of the Ixodida (Acari: Ixodida). Parasites Vectors.

[30] Hebert P.D.N., deWaard J.R., Landry J.-F. (2010). DNA barcodes for 1/1000 of the animal kingdom. Biol. Lett..

[31] Hebert P.D.N., Penton E.H., Burns J.M., Janzen D.H., Hallwachs W. (2004). Ten species in one: DNA barcoding reveals cryptic species in the neotropical skipper butterfly *Astraptes fulgerator*. Proc. Natl. Acad. Sci. USA.

[32] Yadav T., Zhang J.-M., Ouyang J., Leung W., Simoneau A., Zou L. (2022). TERRA and RAD51AP1 promote alternative lengthening of telomeres through an R- to D-loop switch. Mol. Cell.

[33] Ouyang J., Yadav T., Zhang J.-M., Yang H., Rheinbay E., Guo H., Haber D.A., Lan L., Zou L. (2021). RNA transcripts stimulate homologous recombination by forming DR-loops. Nature.

[34] Sharawat S.K., Bakhshi R., Vishnubhatla S., Bakhshi S. (2010). Mitochondrial D-loop variations in paediatric acute myeloid leukaemia: A potential prognostic marker. Br. J. Haematol..

[35] Kim H., Komiyama T., Nitta M., Kawamura Y., Hasegawa M., Shoji S., Orihashi Y., Inomoto C., Kajiwara H., Nakamura N. (2019). D-loop Mutations in Renal Cell Carcinoma Improve Predictive Accuracy for Cancer-Related Death by Integrating with Mutations in the NADH Dehydrogenase Subunit 1 Gene. Genes.

[36] Zhang J., Shang J., Wang F., Huo X., Sun R., Ren Z., Wang W., Yang M., Li G., Gao D. (2022). Decreased mitochondrial D-loop region methylation mediates an increase in mitochondrial DNA copy number in CADASIL. Clin. Epigenet..

[37] Kuang Y., Peng C., Dong Y., Wang J., Kong F., Yang X., Wang Y., Gao H. (2022). NADH dehydrogenase subunit 1/4/5 promotes survival of acute myeloid leukemia by mediating specific oxidative phosphorylation. Mol. Med. Rep..

[38] Velazquez-Villarreal E.I., Maheshwari S., Sorenson J., Fiddes I.T., Kumar V., Yin Y., Webb M.G., Catalanotti C., Grigorova M., Edwards P.A. (2020). Single-cell sequencing of genomic DNA resolves sub-clonal heterogeneity in a melanoma cell line. Commun. Biol..

[39] Shao C., Sun S., Liu K., Wang J., Li S., Liu Q., Deagle B.E., Seim I., Biscontin A., Wang Q. (2023). The enormous repetitive Antarctic krill genome reveals environmental adaptations and population insights. Cell.

[40] Fernández F., Guerrero R.J., Sánchez-Restrepo A.F. (2021). Systematics and diversity of Neotropical ants. Rev. Colomb. De Entomol..

[41] Steiner F.M., Csősz S., Markó B., Gamisch A., Rinnhofer L., Folterbauer C., Hammerle S., Stauffer C., Arthofer W., Schlick-Steiner B.C. (2018). Turning one into five: Integrative taxonomy uncovers complex evolution of cryptic species in the harvester ant Messor “structor”. Mol. Phylogenet. Evol..

[42] Delsinne T., Mackay W., Wild A., Roisin Y., Leponce M. (2012). Distribution and Diversity of the Cryptic Ant Genus *Oxyepoecus* (Hymenoptera: Formicidae: Myrmicinae) in Paraguay with Descriptions of Two New Species. Psyche.

[43] Gaikwad S.S., Ghate H.V., Ghaskadbi S.S., Patole M.S., Shouche Y.S. (2012). DNA barcoding of nymphalid butterflies (Nymphalidae: Lepidoptera) from Western Ghats of India. Mol. Biol. Rep..

[44] Miyata M.N., Kageyama D., Nomura M. (2020). Multiplex PCR for identification of two butterfly sister species: Eurema mandarina and Eurema hecabe. BMC Res. Notes.

[45] Villalta I., Ledet R., Baude M., Genoud D., Bouget C., Cornillon M., Moreau S., Courtial B., Lopez-Vaamonde C. (2021). A DNA barcode-based survey of wild urban bees in the Loire Valley, France. Sci. Rep..

[46] Tyagi K., Kumar V., Kundu S., Pakrashi A., Prasad P., Caleb J.T.D., Chandra K. (2019). Identification of Indian Spiders through DNA barcoding: Cryptic species and species complex. Sci. Rep..

[47] Oh J.-H., Kim S., Lee S. (2022). DNA barcodes reveal population-dependent cryptic diversity and various cases of sympatry of Korean leptonetid spiders (Araneae: Leptonetidae). Sci. Rep..

[48] Bingpeng X., Heshan L., Zhilan Z., Chunguang W., Yanguo W., Jianjun W. (2018). DNA barcoding for identification of fish species in the Taiwan Strait. PLoS ONE.

[49] Scheffers B.R., Oliveira B.F., Lamb I., Edwards D.P. (2019). Global wildlife trade across the tree of life. Science.

[50] Coghlan M.L., Haile J., Houston J., Murray D.C., White N.E., Moolhuijzen P., Bellgard M.I., Bunce M. (2012). Deep Sequencing of Plant and Animal DNA Contained within Traditional Chinese Medicines Reveals Legality Issues and Health Safety Concerns. PLoS Genet..

[51] Newmaster S.G., Grguric M., Shanmughanandhan D., Ramalingam S., Ragupathy S. (2013). DNA barcoding detects contamination and substitution in North American herbal products. BMC Med..

[52] Liu B., Yang J.-W., Liu B.-S., Zhang N., Guo L., Guo H.-Y., Zhang D.-C. (2022). Detection and identification of marine fish mislabeling in Guangzhou’s supermarkets and sushi restaurants using DNA barcoding. J. Food Sci..

[53] van Ruth S.M., van der Veeken J., Dekker P., Luning P.A., Huisman W. (2020). Feeding fiction: Fraud vulnerability in the food service industry. Food Res. Int..

[54] Chen S., Pang X., Song J., Shi L., Yao H., Han J., Leon C. (2014). A renaissance in herbal medicine identification: From morphology to DNA. Biotechnol. Adv..

[55] Marçais G., Kingsford C. (2011). A fast, lock-free approach for efficient parallel counting of occurrences of k-mers. Bioinformatics.

[56] Li S.-Y., Cheng Q.-X., Liu J.-K., Nie X.-Q., Zhao G.-P., Wang J. (2018). CRISPR-Cas12a has both cis- and trans-cleavage activities on single-stranded DNA. Cell Res..

[57] Langmead B., Trapnell C., Pop M., Salzberg S.L. (2009). Ultrafast and memory-efficient alignment of short DNA sequences to the human genome. Genome Biol..

[58] Bae S., Park J., Kim J.-S. (2014). Cas-OFFinder: A fast and versatile algorithm that searches for potential off-target sites of Cas9 RNA-guided endonucleases. Bioinformatics.

