# Supplementary material for: Analysis of Whole-Genome as a Novel Strategy for Animal Species Identification
Source: Int J Mol Sci. 2024 Mar 3;25(5):2955. doi: 10.3390/ijms25052955 (PMC10932323; doi:10.3390/ijms25052955)
Supplement: Supplementary file 1 [file ijms-25-02955-s001.zip › ijms-2864370-supplementary.pdf]

Supplementary legends

Supplementary Table S1 to S6

Supplementary Table S1. the results of bioinformatics analysis for mining species-specific sequences in genomes of *Cervus nippon*, *Cervus elaphus*, and *Rangifer tarandus*.

Supplementary Table S2. primers and PCR amplification program of each species-specific target sequence.

Supplementary Table S3. sample information of fresh animal samples.

Supplementary Table S4. off targets prediction of Cn_target1 in the genomes of *Cervus nippon*, *Cervus elaphus*, and *Rangifer tarandus*.

Supplementary Table S5. off targets prediction of Cn_target2 in the genomes of *Cervus nippon*, *Cervus elaphus*, and *Rangifer tarandus*.

Supplementary Table S6. sample information of commercially available antler products.

Supplementary Figure S1 to S2

Supplementary Figure S1. AGE accurately and specifically identified *Cervus elaphus*, *Cervus nippon*, and *Rangifer tarandus* in fresh animal samples with Sanger sequencing platform.

Supplementary Figure S2. through the CRISPR-Cas12a system, AGE identified *Cervus elaphus*, *Cervus nippon*, and *Rangifer tarandus* in fresh animal samples based on Ce_target1 and -2, Cn_target1 and -2 and Rt_target1 and -2.

Supplementary File S1

Supplementary File S1. Codes used in the bioinformatics analysis steps.

Supplementary Information

Table S1. The results of bioinformatics analysis for mining species-specific sequences in genomes of *Cervus nippon*, *Cervus elaphus*, and *Rangifer tarandus*.

Species	Genome	Fragment sequence	Candidate fragment sequences	Specific sequences
Cervus nippon	nuclear	3,447,186,722	158,287,562	7670053
	mitochondrion	16,410	645	87
Cervus elaphus	nuclear	2,090,198,320	94,378,541	570878
	mitochondrion	16,332	657	103
Rangifer tarandus	nuclear	2,108,086,750	95,321,027	8446736
	mitochondrion	16,338	700	377

Table S2. Primers and PCR amplification program of each species-specific target sequence.

Specific target sequence	Primer	Primer sequence (5'→3')	PCR amplification program
Ce_target1	Ce1F	GCATTAGAGGACTGCTGCC	95℃ 5min<br>94℃ 30s, 60℃ 30s, 72℃ 30s, 35 cycles<br>72℃ 7min
	Ce1R	TGGAGTTCTAAGCTGGTCAC	
Ce_target2	Ce2F	CCTCAGTGTACTGTTCAGAGAT	95℃ 5min<br>94℃ 30s, 56℃ 30s, 72℃ 30s, 35 cycles<br>72℃ 7min
	Ce2R	CATACAGAGTCCTCATGAATGAG	
Ce_target3	Ce3F	CTACTCCAACCTATTGCAGA	95℃ 5min<br>94℃ 30s, 58℃ 30s, 72℃ 1 min, 35 cycles<br>72℃ 7min
	Ce3R	TTTGTGGAGGGATGCTTGAT	
Cn_target1	Cn1F	AGCTGTAACCTGATGCTGATG	95℃ 5min<br>94℃ 30s, 56℃ 30s, 72℃ 30s, 35 cycles<br>72℃ 7min
	Cn1R	GCAACTTGTTTTCCTACCATT	

		TGC	
Cn_target2	Cn2F	AGACTTGAGGAAAGAATGTTCTTTC	95°C 5min<br>94°C 30s, 54°C 30s, 72°C 30s, 35 cycles<br>72°C 7min
	Cn2R	CCAGTGTGAACAAGCATTTACTTAC	
Cn_target3	Cn3F	TACAGCTTTCTACTCAACAC	95°C 5min<br>94°C 30s, 56°C 30s, 72°C 90s, 35 cycles<br>72°C 7min
	Cn3R	CCACAGTTATGTGTGAGCAT	
Rt_target1	Rt1F	AGGCACGTGACTGGTTTATTAGG	95°C 5min<br>94°C 30s, 58°C 30s, 72°C 30s, 35 cycles<br>72°C 7min
	Rt1R	CAGTCCTGCAGCTAAACCTCTG	
Rt_target2	Rt2F	GTGCTTGCTTCTTGGCATCC	95°C 5min<br>94°C 30s, 58°C 30s, 72°C 30s, 35 cycles<br>72°C 7min
	Rt2R	CAGGCAGATTCTTTAGCACTGC	
Rt_target3	Rt3F	GGTCAACAAATCATAAAGATATTGG	94°C 1min<br>94°C 1min, 45°C 90s, 72°C 90s, 5 cycles<br>94°C 1min, 50°C 90s, 72°C 1min, 35 cycles<br>72°C 5min
	Rt3R	TAAACTTCAGGGTGACCAAAAAATCA	
Pa_target1	Pa1F	AATATAAAACCCCCTGCCATTTCACA	95°C 5min<br>94°C 30s, 58°C 30s, 72°C 30s, 35 cycles<br>72°C 7min
	Pa1R	GTAATTCCGGCTGCTAGTACAGG	

Table S3. Sample information of fresh animal samples.

No.	Sample type	Market label	Locality	Barcode ID
1	Fresh sample	Red deer antler	Changchun, Jilin	*Cervus elaphus*
2	Fresh sample	Red deer antler	Changchun, Jilin	*Cervus elaphus*
3	Fresh sample	Red deer meat	Changchun, Jilin	*Cervus elaphus*

4	Fresh sample	Red deer meat	Changchun, Jilin	*Cervus elaphus*
5	Fresh sample	Sika deer antler	Fushun, Liaoning	*Cervus nippon*
6	Fresh sample	Sika deer antler	Changchun, Jilin	*Cervus nippon*
7	Fresh sample	Sika deer meat	Changchun, Jilin	*Cervus nippon*
8	Fresh sample	Reindeer antler	Changchun, Jilin	*Rangifer tarandus*
9	Fresh sample	Reindeer antler	Changchun, Jilin	*Rangifer tarandus*
10	Fresh sample	Reindeer antler	Changchun, Jilin	*Rangifer tarandus*
11	Fresh sample	Reindeer antler	Changchun, Jilin	*Rangifer tarandus*

Table S4. Off targets prediction of Cn_target1 in the genomes of *Cervus nippon, Cervus elaphus,* and *Rangifer tarandus.*

Species	Number of mismatches					
	0	1	2	3	4	5
Cervus nippon	0	1	0	0	3	48
Cervus elaphus	0	0	0	0	4	63
Rangifer tarandus	0	0	0	0	7	65

Table S5. Off targets prediction of Cn_target2 in the genomes of *Cervus nippon, Cervus elaphus,* and *Rangifer tarandus.*

Species	Number of mismatches					
	0	1	2	3	4	5
Cervus nippon	1	0	0	0	3	35
Cervus elaphus	0	0	0	0	4	34
Rangifer tarandus	0	0	0	0	3	38

Table S6. Sample information of commercially available antler products.

1	commercially available products	Antler powder	Hebei	*Cervus nippon*
2	commercially available products	Sika deer antler powder	Kunming, Yunnan	*Rangifer tarandus*
3	commercially available products	Sika deer antler powder	Shuangyang, Jilin	*Rangifer tarandus*
4	commercially available products	Antler powder	Yinchuan, Ningxia	*Cervus elaphus*
5	commercially available products	Antler tablet	Nanjing, Jiangsu	*Cervus elaphus*
6	commercially available products	Antler powder	Nanjing, Jiangsu	*Cervus elaphus*
7	commercially available products	Red deer antler tablet	Luoyang,Henan	*Rangifer tarandus*
8	commercially available products	Antler tablet	Bozhou, Anhui	*Rangifer tarandus*
9	commercially available products	Antler tablet	Bozhou, Anhui	*Rangifer tarandus*
10	commercially available products	Antler powder	Hebei	*Cervus nippon*
11	commercially available products	Antler powder	Liaoning	*Rangifer tarandus*
12	commercially available products	Antler tablet	Bozhou, Anhui	*Rangifer tarandus*
13	commercially available products	Antler powder	Bozhou, Anhui	*Rangifer tarandus*
14	commercially available products	Red deer antler powder	Beijing	*Cervus elaphus*

15	commercially available products	Sika deer antler tablet	Changchun, Jilin	*Cervus elaphus*
16	commercially available products	Red deer antler powder	Wushun, Liaoning	*Cervus elaphus*
17	commercially available products	Antler powder	Wuhan, Hubei	*Cervus elaphus*
18	commercially available products	Antler powder	Kunming, Yunnan	*Rangifer tarandus*
19	commercially available products	Antler powder	Changchun, Jilin	*Cervus nippon*
20	commercially available products	Antler tablet	Bozhou, Anhui	*Rangifer tarandus*
21	commercially available products	Antler tablet	Dunhua, Jilin	*Rangifer tarandus*
22	commercially available products	Antler powder	Luzhou, Sichuan	*Rangifer tarandus*
23	commercially available products	Antler tablet	Liwan, Guangdong	*Rangifer tarandus*
24	commercially available products	Sika deer antler powder	Tonghua	*Cervus elaphus*
25	commercially available products	Antler tablet	Kunming, Yunnan	*Rangifer tarandus*
26	commercially available products	Antler tablet	Jiangmen, Guangdong	*Rangifer tarandus*
27	commercially available products	Antler powder	Kunming, Yunnan	*Rangifer tarandus*
28	commercially available products	Antler tablet	Wenshan, Yunnan	*Cervus elaphus*
29	commercially	Antler tablet	Daxing, Beijing	*Cervus elaphus*

	available products			
30	commercially available products	Antler tablet	Shanghai	*Cervus elaphus*
31	commercially available products	Antler powder	Changchun, Jilin	*Rangifer tarandus*
32	commercially available products	Antler powder	Kunming, Yunnan	*Rangifer tarandus*
33	commercially available products	Antler powder	Changchun, Jilin	*Rangifer tarandus*
34	commercially available products	Red deer antler	Wushun, Liaoning	*Cervus elaphus*
35	commercially available products	Red deer antler	Wushun, Liaoning	*Cervus elaphus*

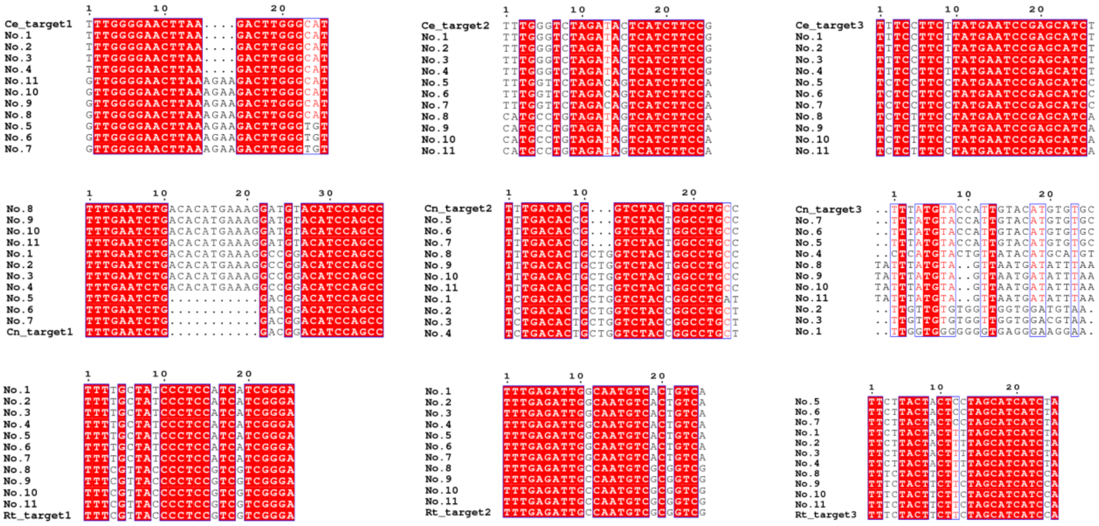

Figure S1 AGE accurately and specifically identified *Cervus elaphus*, *Cervus nippon*, and *Rangifer tarandus* in fresh animal samples with Sanger sequencing platform. No.1, No.2, No.3, and No.4: fresh *Cervus elaphus* samples; No.5, No.6, and No.7: fresh *Cervus nippon* samples; No.8, No.9, No.10, and No.11: fresh *Rangifer tarandus* samples.

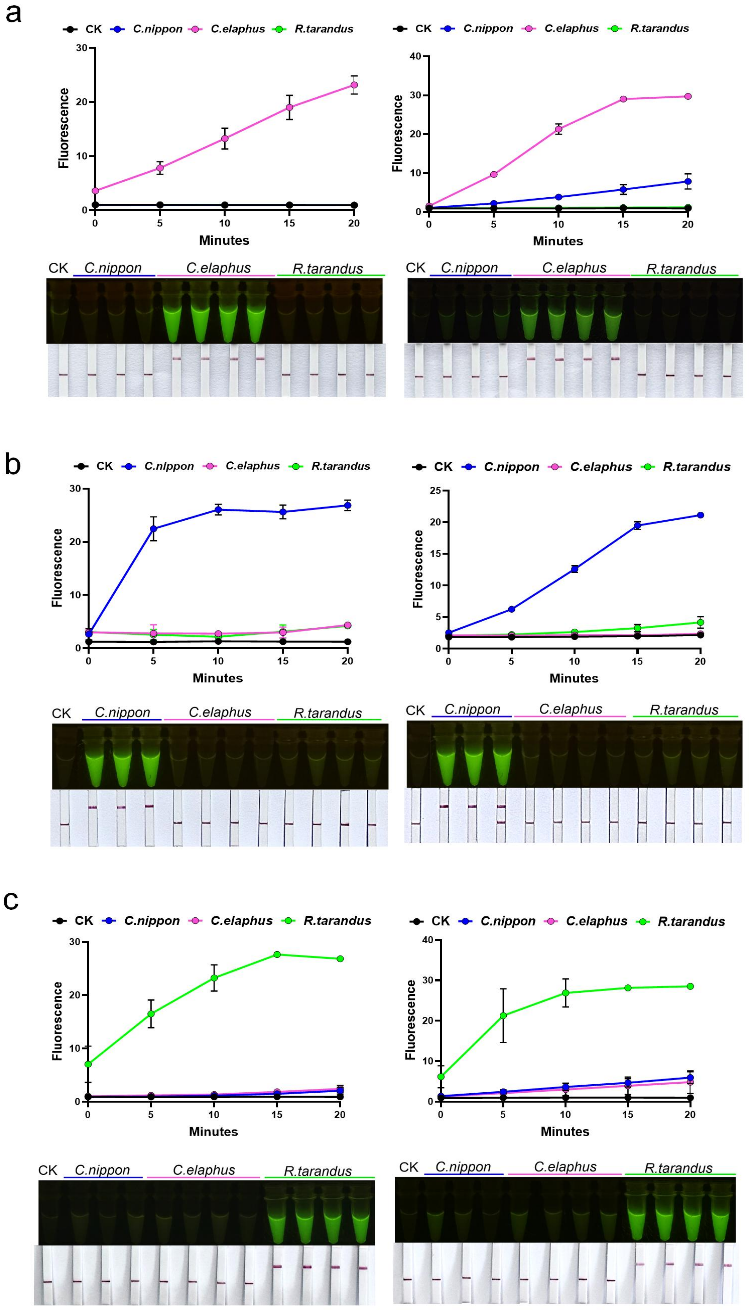

Figure S2 Through the CRISPR-Cas12a system, AGE identified *Cervus elaphus*, *Cervus nippon*, and *Rangifer tarandus* in fresh animal samples based on Ce_target1, 2, Cn_target1, 2 and Rt_target1, 2. (a) The identification of *Cervus elaphus* in fresh animal samples using Ce_target1, 2. CK: nuclease-free water. (b) The identification of *Cervus nippon* in fresh animal samples using Cn_target1, 2. CK: nuclease-free water. (a) The identification of *Rangifer tarandus* in fresh animal samples using Rt_target1, 2. CK: nuclease-free water.

File S1. Codes used in the bioinformatics analysis steps.

#The genomes were cut into K bp fragments using Jellyfish to generate kmers.

/path/jellyfish count -C -m k -s 3G -t 6 -o x_kmer x_genome.fa # k, length of mer; x, one species

/path/jellyfish dump -c -t x_kmer > x_kmer.stat

#The kmers with PAM (TTTV starting or VAAA ending) sequences were extracted and compared to their genomes.

awk '$1~/^TTTA/||$1~/^TTTG/||$1~/^TTTC/||$1~/GAAA$/||$1~/CAAA$/||$1~/TAAA$/{print ">"NR"_"$2"\n"$1}' x_kmer.stat > x_kmer_PAM.fa

/path/bowtie-1.1.0/bowtie-build x_genome.fa x_genome.fa

/path/bowtie-1.1.0/bowtie -f -v 0 -p 8 x_genome.fa x_kmer_PAM.fa x_kmer_PAM_ genome.bwt

#The candidate fragment sequences of one species were mapped to these of two other species and only candidate fragment sequences with sequences differences of three or more mismatches and all indels were retained.

sed '$!N;s/\n/\t/' x_kmer_PAM.fa|awk '$2~/^TTTA/||$2~/^TTTG/||$2~/^TTTC/{print $1"\n"substr($2,5,21)}' > x_kmer_PAM_21_TTT.fa

sed '$!N;s/\n/\t/' x_kmer_PAM.fa|awk '$2~/GAAA$/||$2~/CAAA$/||$2~/TAAA$/{print $1"\n"substr($2,1,21)}' > x_kmer_PAM_21_AAA.fa

/path/bowtie-1.1.0/bowtie-build x_kmer_PAM_21_AAA.fa x_kmer_PAM_21_AAA.fa

/path/bowtie-1.1.0/bowtie-build x_kmer_PAM_21_TTT.fa x_kmer_PAM_21_TTT.fa

```
/path/bowtie-1.1.0/bowtie -f -v 2 -p 8 --norc b_kmer_PAM_21_TTT.fa
a_kmer_PAM_21_TTT.fa a_kmer_PAM_21_TTT_b.bwt #a, target species; b,
other species
```

```
/path/bowtie-1.1.0/bowtie -f -v 2 -p 8 --norc b_kmer_PAM_21_AAA.fa
a_kmer_PAM_21_AAA.fa a_kmer_PAM_21_AAA_b.bwt
```

```
cat a_kmer_PAM_21_TTT_b.bwt a_kmer_PAM_21_AAA_b.bwt |awk '{print ">"
$1}' |sort -u |awk 'FNR==NR{a[$1]=$0;next}{print $0"\t"a[$1]}' - a_kmer_PAM.
fa |sed '$!N;s/\n/\t/'| awk '$2!~/>/ {print$1"\n"$2}' > a_kmer_PAM_uniq.fa
```
